# The potential role of biomarkers CD28 and PF4 in the pathogenesis of idiopathic pulmonary fibrosis and their impact on the prognosis: an immune microenvironment analysis

**DOI:** 10.1186/s41065-025-00464-x

**Published:** 2025-06-07

**Authors:** Li Yan, Jiang-Han Li, Ai-Li Zhang, He Li, Bo Pang, De-Yang Meng, Qian Fu, Li-Juan Du, Yan Su

**Affiliations:** 1 Department of Pulmonary and Critical Care Medicine, Hebei General Hospital, No. 348 of Heping West Road, Xinhua District, Shijiazhuang, 050051 Hebei Province China; 2 Department of Graduate College, Hebei Medical University, Shijiazhuang, 050000 Hebei Province China

**Keywords:** CD28, Idiopathic pulmonary fibrosis, Mitochondrion, PF4, Programmed cell death

## Abstract

**Background:**

This study aims to identify and investigate biomarkers associated with mitochondrial-related genes (MRGs) and programmed cell death-related genes (PCDRGs) that concurrently influence the progression of idiopathic pulmonary fibrosis (IPF) and to explore the underlying biological mechanisms involved.

**Methods:**

The GSE28042 and GSE27957 datasets, comprising 1,136 MRGs and 1,548 PCDRGs, were utilized in this study. Differentially expressed genes (DEGs) between the IPF and control groups were initially identified through differential expression analysis. Subsequently, key module genes closely associated with IPF samples were selected using Weighted Gene Co-expression Network Analysis (WGCNA). Intersection genes 1 and 2 were then identified by overlapping DEGs with key module genes, MRGs, and PCDRGs. Candidate genes were further selected through Spearman correlation analysis involving intersection genes 1 and 2. Additionally, biomarkers were identified, and a risk model was developed using Cox regression analysis, proportional hazards (PH) assumption testing, and machine learning methods. Patients with IPF were stratified into high- and low-risk cohorts. Finally, functional enrichment analysis, immune infiltration analysis, regulatory network construction, and reverse transcription quantitative PCR (RT-qPCR) were conducted separately to validate the findings.

**Results:**

CD28 and PF4 were identified as biomarkers, and a risk model was established. The distinct risk cohorts exhibited differences in pathways related to hemostasis, prion diseases, and other biological processes. A significant positive correlation with was observed between CD28 and native CD4 T cells, while PF4 showed a negative correlation with activated NK cells. Based on these two biomarkers, 30 miRNAs and 532 lncRNAs were predicted, resulting in the construction of a lncRNA–miRNA–biomarker network. Additionally, 11 chemicals associated with these biomarkers were identified. RT-qPCR analysis further confirmed that expression levels of CD28 and PF4 were significantly reduced in IPF samples (*P* < 0.05).

**Conclusion:**

The results of this study suggested that the biomarkers CD28 and PF4 might play a potential role in the pathogenesis of IPF and might have an impact on the prognosis of the disease. These findings might offer valuable insights for future treatment strategies and prognostic evaluation for patients with IPF.

**Supplementary Information:**

The online version contains supplementary material available at 10.1186/s41065-025-00464-x.

## Background

Idiopathic pulmonary fibrosis (IPF) is characterized by diffuse alveolitis and structural abnormalities in the alveoli, ultimately resulting in pulmonary interstitial fibrosis [1]. The incidence and mortality rates of IPF increase significantly with age, with a prevalence reaching up to 94 cases per 100,000 individuals aged 65 years and older [2–4]. IPF is a progressive disease characterized by a poor prognosis, with survival rates comparable to those of advanced cancers. The median survival time after diagnosis is approximately 3 to 4 years [5]. IPF results from sustained or recurrent lung epithelial injury, followed by the activation of fibroblasts and differentiation into myofibroblasts. The persistent myofibroblast phenotype leads to excessive deposition of the extracellular matrix (ECM) and abnormal lung repair, causing tissue scarring, distortion of alveolar structure, and irreversible loss of lung function [6]. Despite ongoing research, the etiology and pathogenesis of IPF remain only partially understood, involving complex mechanisms such as aging and a number of changes with aging including telomere attrition, cell senescence, and mitochondrial dysfunction, environmental exposures, epithelial cell injury, and endoplasmic reticulum stress [7]. Reactive oxygen species (ROS) from NADPH oxidase (NOX) or mitochondria may also contribute to IPF pathology [8].

Currently, pirfenidone and nintedanib have shown efficacy in slowing the decline in pulmonary function, however, their ability to halt disease progression and enhance quality of life in patients remains limited [9, 10]. Although lung transplantation can improve quality of life and extend survival, its widespread use is constrained by high technical demands and financial costs. Consequently, there is an urgent need to identify novel biomarkers for early intervention, explore exposure-related factors, and discover therapeutic targets essential for improving IPF management and patient outcomes [11, 12]. ERS-related biomarkers have been identified in IPF, highlighting SPP1 and M2 macrophages, and the corresponding diagnostic and prognostic models offer strong predictive capabilities, unveiling new therapeutic avenues [13]. ABHD5 may also serve as a potential biomarker, where low ABHD5 expression could potentially accelerate the progression of pulmonary fibrosis [14].

Programmed cell death (PCD) is a genetically regulated and orderly process essential for organismal development. This process occurs through various mechanisms, including apoptosis, anoikis, autophagy, alkaliptosis, cuproptosis, entosis, entotic cell death, immunogenic cell death, ferroptosis, methuosis, necroptosis, NETosis, netotic cell death, oxeiptosis, pyroptosis, parthanatos, lysosome-dependent cell death, and paraptosis. Collectively, these mechanisms play a critical role in maintaining tissue homeostasis and eliminating damaged cells [15]. Mitochondria are integral to this process by mitigating oxidative stress, regulating signaling pathways, and controlling PCD [16]. Disruption of mitochondrial morphology and structural integrity influences the release of pro-apoptotic factors from mitochondria. Additionally, mitochondrial dysfunction, impaired oxidative phosphorylation, and reduced ATP production induce cellular stress, triggering PCD pathways [17, 18].

Emerging evidence suggests that PCD processes, such as proliferation, apoptosis, senescence, and autophagy, contribute to IPF pathogenesis [19]. The senescence of lung fibroblasts in IPF reduces apoptotic activity, while the development of a senescence-associated secretory phenotype (SASP) may play a pivotal role in fibrosis progression [20]. Furthermore, mitochondrial dysfunction is a hallmark of aging-related lung diseases. In IPF, multiple regulatory pathways governing mitochondrial function are disrupted in epithelial cells, fibroblasts, and macrophages, impairing stress responses and creating a vulnerable environment conducive to fibrosis [21, 22]. To date, the interaction between mitochondrial genes and those involved in PCD remains poorly understood in IPF, and the precise mechanisms underlying their combined effects in this disease are yet to be elucidated.

In this study, differentially expressed genes (DEGs) between IPF and normal samples were identified using transcriptomic analysis, and key module genes were selected through Weighted Gene Co-expression Network Analysis (WGCNA). Based on these findings, the roles of mitochondrial- and programmed cell death-related genes in the pathogenesis of IPF, as well as their association with immune infiltration, referring to the deconvolution of transcriptomic data to identify the composition and interactions of immune cells within the tissue microenvironment, were explored. Additionally, two relevant biomarkers were identified as potential indicators for IPF prognosis.

## Materials and methods

### Data extraction

The GSE28042 and GSE27957 datasets related to IPF were retrieved from the Gene Expression Omnibus (GEO) (https://www.ncbi.nlm.nih.gov/gds). The GSE28042 dataset comprised 75 peripheral blood mononuclear cells (PBMCs) from patients with IPF and 19 PBMCs from control samples, utilizing the GPL6480 platform (Supplementary Table 1). The GSE27957 dataset included 45 PBMCs from IPF samples based on the GPL5175 platform, and 42 IPF samples with survival data were selected for subsequent analysis (Supplementary Table 2). A total of 1,136 mitochondrial-related genes (MRGs) were obtained from the MitoCarta 3.0 database (http://www.broadinstitute.org/mitocarta), while 1,548 programmed cell death-related genes (PCDRGs) were sourced from existing literature [16].

### Differential expression analysis and weighted gene Co-expression network analysis (WGCNA)

DEGs in GSE28042 (IPF vs. control) were identified using the “limma” R package (v 3.52.4) initially, applying the screening criteria of *P* < 0.05 and |log2 Fold Change (FC)| ≥ 0.50 [23]. The DEGs were then visualized using a volcano plot and heatmap, created with the “ggplot2” (v 3.3.6) and “pheatmap” (v 1.1.9) R packages, respectively [24, 25]. Subsequently, WGCNA was performed to identify key modules significantly associated with IPF samples, utilizing the “WGCNA” R package (v 1.71) [26].

Initially, the samples were clustered to eliminate outliers. The soft threshold (β) was determined such that the scale-free R² exceeded 0.9, with mean connectivity approaching 0. A co-expression matrix was then constructed, with each gene module required to contain a minimum of 100 genes. Distinct gene modules were identified using different colors. The correlation coefficients between all samples and gene modules were calculated and displayed in a heatmap. Ultimately, crucial modules showing a strong association with IPF samples were selected based on the criteria |cor| > 0.30 and *P* < 0.05. Genes from these key modules were designated as key module genes. The first set of intersection genes was identified by overlapping the DEGs, key module genes, and MRGs, while the second set of intersection genes was selected by overlapping DEGs, key module genes, and PCDRGs.

### Identification and functional analysis of candidate genes

Spearman correlation analysis was conducted to calculate the association coefficients between the first set of intersection genes and the second set of intersection genes, using the “corrplot” R package (v 0.92) [27]. Candidate genes were selected based on the screening criteria of |cor| > 0.70 and adjusted *P* < 0.05. To explore the functional pathways associated with the candidate genes, Gene Ontology (GO) and Kyoto Encyclopedia of Genes and Genomes (KEGG) analyses were performed using the “clusterProfiler” R package (v 4.7.1), with an adjusted *P* < 0.05 [28]. Furthermore, to understand the complex protein interactions between candidate genes and their functional associations and to reveal their potential regulatory mechanisms a protein-protein interaction (PPI) network was constructed using the STRING database (https://string-db.org/). Screening criteria was set to confidence > 0.4.

### Establishment and validation of risk model

Univariate Cox regression analyses (*P* < 0.05) and proportional hazard (PH) hypothesis tests (*P* > 0.05) were performed using the “survival” R package (v 3.4–0) based on information on the clinical characteristics of the disease samples in the training set. The expression of candidate genes was compared with survival time as a continuous variable to predict whether candidate genes were associated with patient survival [29]. Following this, the least absolute shrinkage and selection operator (LASSO) method was applied to further refine gene selection based on the minimum lambda value using the “glmnet” package [30]. After successfully passing the LASSO analysis, multivariate Cox regression analysis (*P* < 0.05) was conducted to identify biomarkers. Subsequently, a risk model was constructed based on these biomarkers. The formula for calculating the risk score is as follows:

$$\:risk\:score = \sum\limits_{i = 1}^n {(coefi * \:Xi)} $$

The “coef” represents the coefficients of the biomarkers, while “x” denotes the expression levels of these biomarkers. Subsequently, to evaluate and validate the risk model, patients with IPF from the GSE28042 and GSE27957 datasets were categorized into high-risk and low-risk groups based on the median value of their risk scores. Furthermore, for these two risk cohorts, risk curves, Kaplan-Meier (K - M) survival curves (*P* < 0.05), and the model’s 1-year, 2-year, and 3-year receiver operating characteristic (ROC) curves (Area Under the Curve [AUC] > 0.6) were plotted separately for the GSE28042 and GSE27957 datasets using the “survival” and “survivalROC” R packages (v 1.0.3.1) [31].

### Clinical characteristics analysis and nomogram establishment

To further explore the relationship between the risk score and clinical characteristics, separate analyses were conducted based on age (< 75 and ≥ 75 years) and sex (female and male) (*P* < 0.05). In addition, risk scores and clinical characteristics (age, sex) were included in one-way Cox regression analyses (*P* < 0.05), PH hypothesis tests were performed based on the results of one-way Cox regression analyses (*P* > 0.05), and traits that passed the PH hypothesis test were subjected to multifactorial Cox regression analyses (*P* < 0.05). This process aimed to identify independent prognostic factors, and a nomogram was constructed using the “survival” and “rms” packages (v 6.3–0) [32]. Subsequently, calibration curves and decision curve analysis (DCA) for 1-year, 2-year, and 3-year outcomes were plotted to assess the accuracy of the nomogram.

### Gene set enrichment analysis (GSEA)

To examine the functional diversity and pathway differences between the two risk cohorts, differentially expressed genes (high risk vs. low risk) were identified and ranked. Additionally, download “c5.go.bp.v2023.2.Hs.symbols.gmt” from the “msigdbr” (v 7.5.1) [33] database as a background gene set, and then subjected to Gene Set Enrichment Analysis (GSEA) (v 1.44.5) [34], including Gene Ontology (GO) and Kyoto Encyclopedia of Genes and Genomes (KEGG). The screening criterion set at an adjusted *P* < 0.05.

### Immune infiltration analysis

CIBERSORTx was a deconvolution tool that could be used for immune cell proportion estimation through deconvolution, enabling the calculation of infiltration scores for 22 immune cell types in IPF samples from the GSE28042 dataset. Immune cells with zero abundance across all samples were excluded, and the results were presented using a stacked diagram. The differences in infiltration scores between high-risk and low-risk cohorts were then analyzed using the Wilcoxon test. Furthermore, Spearman’s correlation analysis was conducted to explore the relationship between the biomarkers and the 22 immune cell types.

### Regulation network analysis

To investigate the expression patterns of biomarkers, miRNAs targeting these biomarkers were predicted, and their intersections were identified using the miRDB (https://mirdb.org/) and TargetScan (https://www.targetscan.org/) databases. Subsequently, lncRNAs targeting the identified miRNAs were predicted through the Starbase database (http://starbase.sysu.edu.cn/) with a pancancerNum ≥ 10. A lncRNA-miRNA-biomarker network was then constructed using Cytoscape software (v 3.8.1) [35]. Additionally, potential chemicals targeting the biomarkers were identified in the Comparative Toxicogenomics Database (CTD) (https://ctdbase.org/), applying a threshold of reference counts greater than 2. Finally, the biomarker-chemical network was also established using Cytoscape software.

### Reverse transcription quantitative PCR (RT-qPCR)

A total of 10 samples, comprising 5 normal and 5 IPF peripheral blood mononuclear cell samples, were collected from patients at Hebei General Hospital. Informed consent was obtained from all participants, and all research procedures adhered to the principles outlined in the Declaration of Helsinki. The study received approval from the Ethics Committee of Hebei General Hospital (approval number: NO.2024-029). Total RNA was extracted from the samples using TRIzol reagent (Ambion, USA) in accordance with the manufacturer’s instructions. RNA concentration was assessed using a NanoPhotometer N50. Complementary DNA (cDNA) was synthesized via reverse transcription with the SureScript First-Strand cDNA Synthesis Kit, conducted using the S1000™ Thermal Cycler (Bio-Rad, USA). The sequences of all primers are detailed in Supplementary Table 1. The qPCR assay was performed using the CFX Connect Real-Time Quantitative Fluorescence PCR Instrument (Bio-Rad, USA) with the following cycling conditions: pre-denaturation at 95 °C for 1 min, denaturation at 95 °C for 20 s, annealing at 55 °C for 20 s, and extension at 72 °C for 30 s, totaling 40 cycles. Relative quantification of mRNAs was calculated using the 2^−ΔΔCT^ method.

### Statistical analysis

Statistical analyses were performed using R (v 4.2.2). Differences were evaluated using the Wilcox test, with a significance threshold set at *P* < 0.05.

## Results

### Identification of intersection genes

**Fig. 1:**
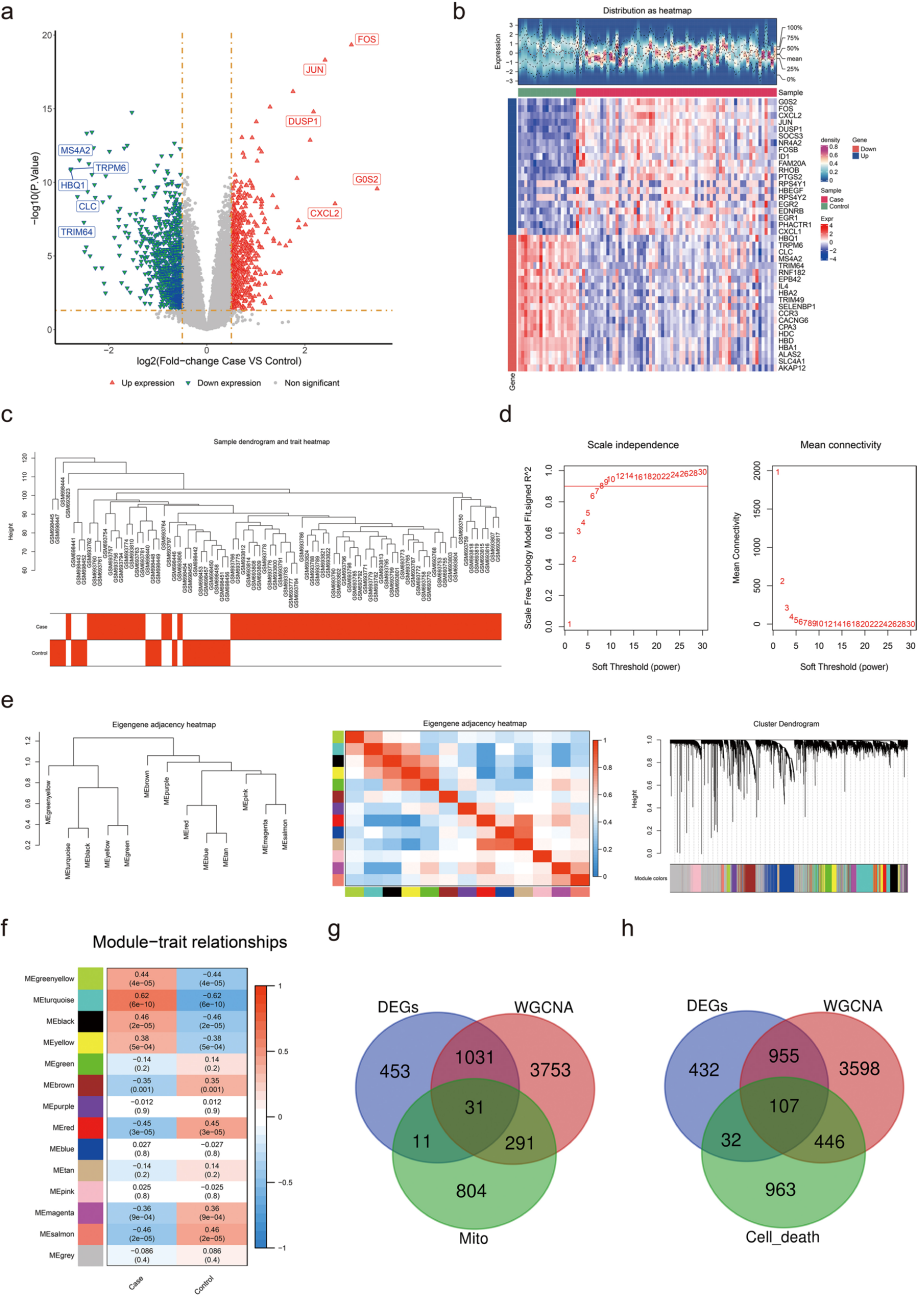
Screening process for DEGs in the GSE28042 dataset. Panel **(a)**: Volcano plots depicting the DEGs identified in the dataset. Panel **(b)**: Heatmap of the top 20 DEGs, with red indicating high expression and blue indicating low expression. Panel **(c)**: Sample cluster tree displaying the clustering of patients with IPF and control subjects. Panel **(d)**: Soft threshold screening revealing the optimal soft threshold for WGCNA, determined to be 9. The horizontal axis represents the weight parameter power values, while the left vertical axis indicates the scale-free fit index (signed R²), where a higher squared correlation coefficient signifies that the network more closely resembles a scale-free distribution. The right vertical axis represents the mean of all gene adjacency functions within the corresponding gene module. Panel **(e)**: Establishment of a co-expression network identifying 14 gene modules. Panel **(f)**: Heatmap showing the correlation between these modules and various traits. Panel **(g)**: Identification of 31 intersection genes through the overlap of DEGs, key module genes, and MRGs. Panel **(h)**: Identification of 107 intersection genes by overlapping DEGs, key module genes, and PCDRGs

A total of 1,526 DEGs were identified in the comparison between IPF and control samples, comprising 584 upregulated DEGs and 942 downregulated DEGs (Fig. 1a-b). The maximum height of the sample cluster tree was set at 105, resulting in the removal of outlier samples (Fig. 1c). The soft threshold (β) was determined to be 9, which first exceeded the threshold indicated by the red line (0.9), while the mean connectivity approached 0 (Fig. 1d). A co-expression matrix was then constructed, resulting in the identification of 14 gene modules, each displayed in distinct colors (Fig. 1e). The modules MEgreenyellow, MEturquoise, MEblack, MEyellow, MEbrown, MEred, MEmagenta, and MEsalmon showed strong associations with IPF, meeting the criteria of (|cor| > 0.30, *P* < 0.05) (Fig. 1f). A total of 5,106 genes from these key modules were designated as key module genes. Ultimately, 31 intersection genes (referred to as intersection genes 1) were identified by overlapping the DEGs, key module genes, and MRGs (Fig. 1g), while 107 intersection genes (intersection genes 2) were selected by overlapping the DEGs, key module genes, and PCDRGs (Fig. 1h).

### Identification of candidate genes

**Fig. 2:**
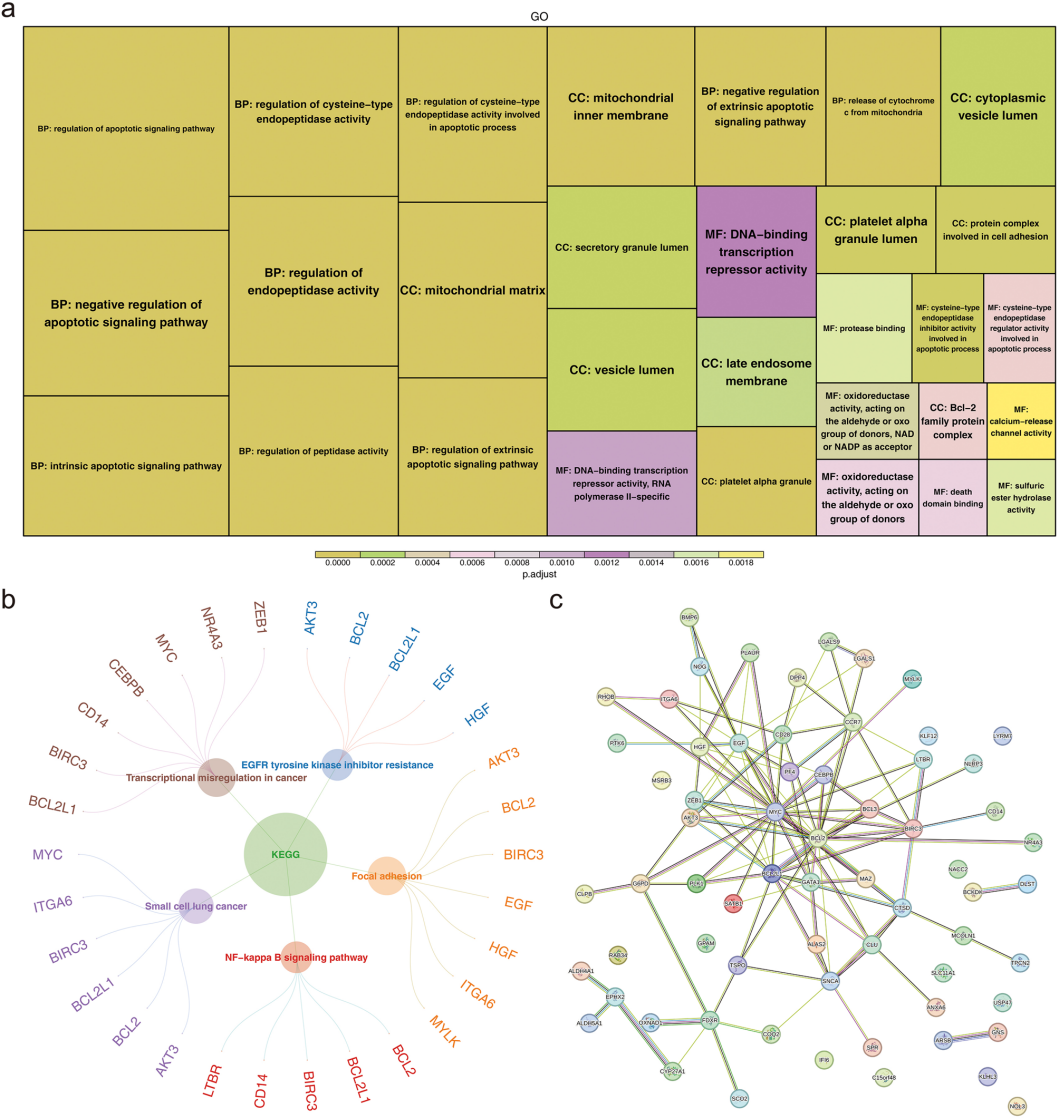
Identification of candidate genes. Panel **(a)**: GO enrichment analysis of the candidate genes. The box size represents the number of included genes and the color indicates the level of significance. Panel **(b)**: KEGG-enriched pathways associated with the candidate genes. Circles denote the enriched pathways, with the outer circles representing the genes associated with each pathway. Panel **(c)**: Protein-protein interaction network of the candidate genes, comprising 117 edges and 54 nodes, highlighting a strong intergene association

Correlation analysis revealed that 66 genes exhibited a strong correlation between intersection genes 1 and intersection genes 2, leading to their designation as candidate genes for subsequent analysis. Additionally, these candidate genes were significantly enriched in 1,045 GO terms, including “mitochondrial matrix” and “DNA-binding transcription repressor activity” (Fig. 2a). Furthermore, the candidate genes were notably associated with 56 KEGG pathways, such as “transcriptional misregulation in cancer” and “focal adhesion,” among others (Fig. 2b). These findings suggestted that these candidate genes might play a role in several aspects of cellular metabolism, regulation of gene expression, and cancer development. Moreover, the protein-protein interaction (PPI) network of the candidate genes revealed 117 nodes and 54 interaction relationships, including interactions such as MYC-CEBPB and BCL2-MAZ (Fig. 2c), these findings provide clues for exploring the mechanisms of candidate genes in disease development.

### Efficacy of the risk model based on 2 biomarkers

**Fig. 3:**
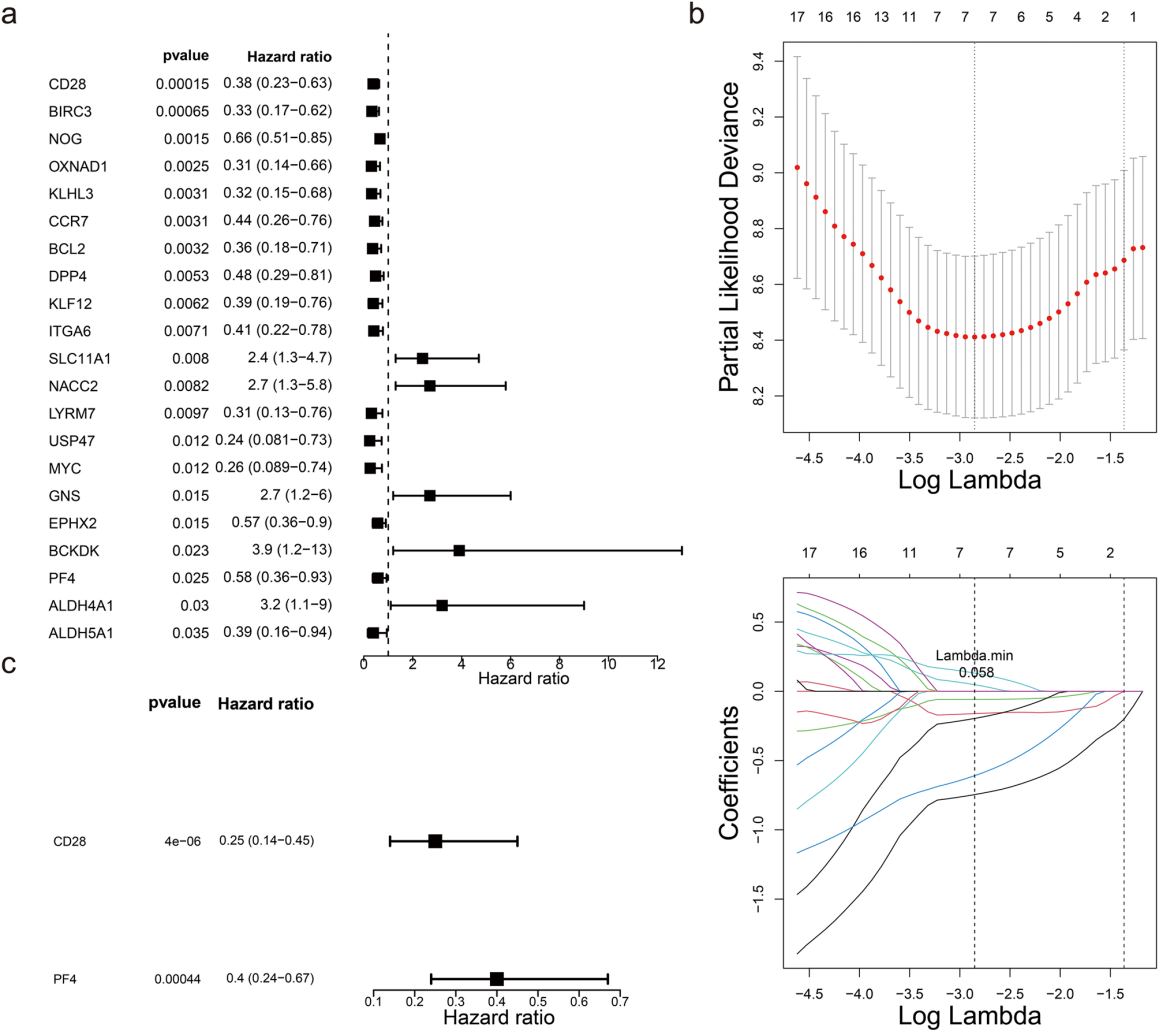
Acquisition of biomarkers. Panel **(a)**: Univariate Cox forest plot identifying 21 genes significantly associated with survival. The first column lists the names of these candidate genes, the second column presents the *p*-values, and the third column shows the hazard ratio (HR) values along with their confidence intervals. The middle point on the right plot represents the HR value, while the horizontal range indicates the confidence interval. Panel **(b)**: LASSO regression analysis conducted on the 21 genes derived from the univariate Cox regression analysis. Panel **(c)**: Multivariate Cox forest plot highlighting two biomarkers identified through this analysis

**Fig. 4:**
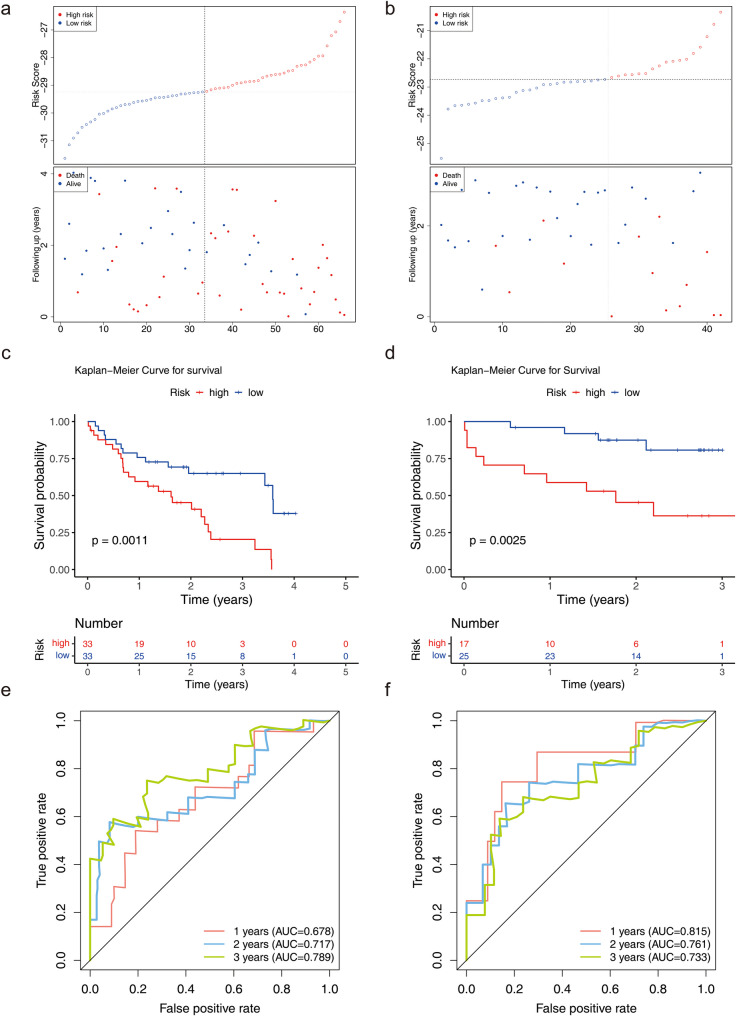
Construction and validation of risk models. Panels **(a)** and **(b)**: Risk curves, for the GSE28042 (left) and GSE27957 (right) datasets, illustrating the distribution of risk scores among patients. Panels **(c)** and **(d)**: Kaplan-Meier curves for GSE28042 (left) and GSE28042 (right) revealing a difference in survival between high- and low-risk patients (*p* < 0.05). Panels **(e)** and **(f)**: ROC curves for GSE28042 (left) and GSE28042 (right), indicating the model’s good predictive performance with AUC values exceeding 0.6 for 1, 2, and 3 years

Using the candidate genes, univariate Cox regression analysis identified 21 genes with a significance level of *P* < 0.05 (Fig. 3a), all of which passed the PH assumption test with *P* > 0.05. This suggests that these genes were significantly correlated with survival status and were potential survival-related genes. Subsequently, LASSO analysis was performed, resulting in the selection of 7 genes at the minimum lambda value of 0.058, which contributed more to survival prediction (Fig. 3b). Multivariate Cox regression analysis further identified 2 biomarkers (CD28 and PF4) with *P* < 0.05 (Fig. 3c). Two biomarkers (CD28 and PF4) remained statistically significantly correlated with patient survival when the effects of other variables were considered, and their effects were independent. Therefore, a risk model for IPF based on these two biomarkers was conducted with the aim of evaluating patients to assess the probability of survival or the risk of death of a patient over a certain period of time. In both datasets, the risk curves indicated a higher number of deceased samples in the high-risk cohort (Fig. 4a-b). Additionally, Kaplan-Meier (K-M) curves demonstrated that high-risk patients exhibited a lower probability of survival (Fig. 4c-d). These results suggested that patients in the high-risk cohort had a poorer prognosis and may have had a shorter survival, and that more attention needs to be paid to this group of patients in future treatment and more aggressive treatment measures considered. The receiver operating characteristic (ROC) curves revealed that the area under the curve (AUC) values in both datasets exceeded 0.6, indicating a strong predictive capability of the model (Fig. 4e-f).

### Risk score as an independent prognostic factor for IPF

**Fig. 5:**
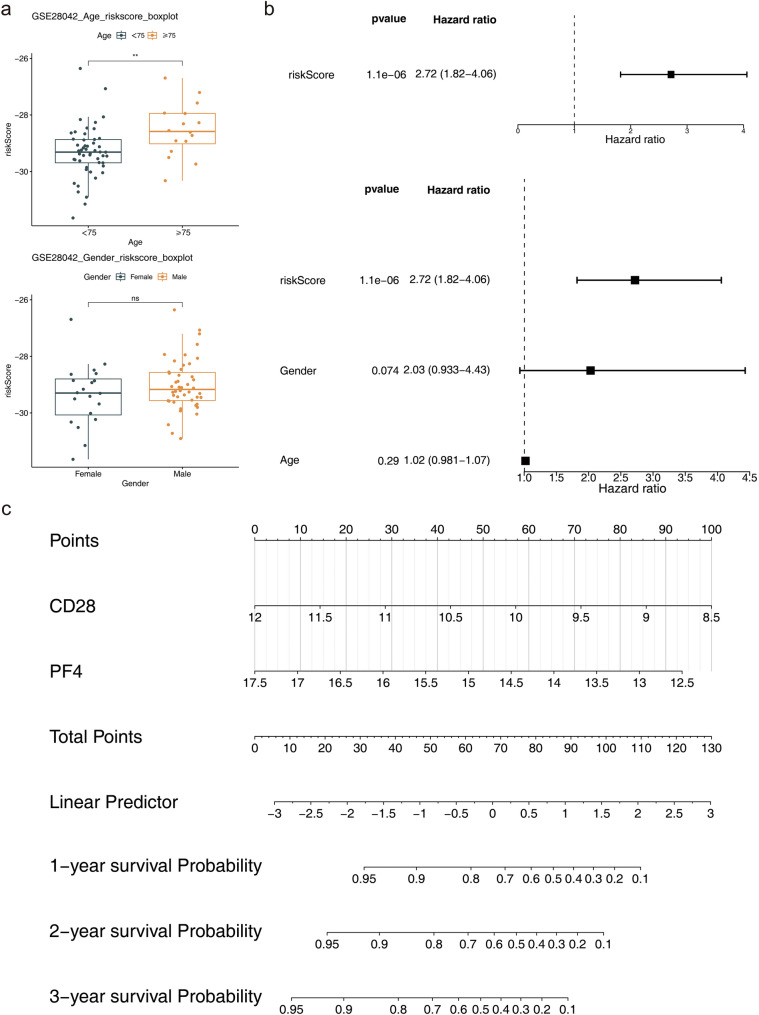
Independent prognostic factors and construction of the nomogram. Panel **(a)**: Displays two factors: age (left) and gender (right). Panel **(b)**: Independent prognostic analysis forest plot, highlighting that the risk score serves as an independent prognostic factor. The left side features a univariate Cox forest plot and the right side presents a multivariate Cox forest plot. Panel **(c)**: Nomogram of biomarkers, indicating that a higher overall score corresponds to a lower survival rate in patients with IPF

**Fig. 6:**
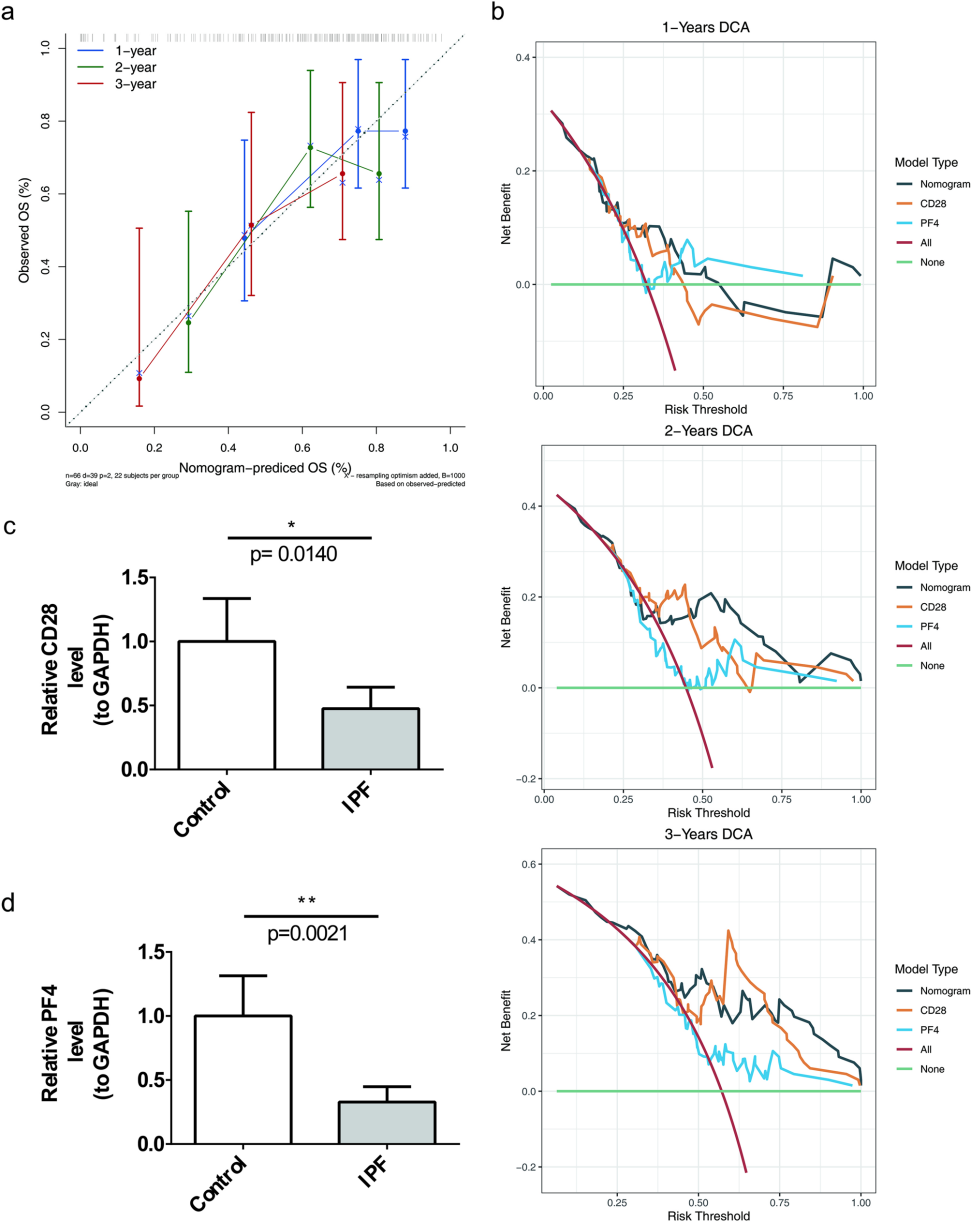
Validation of the predictive power of nomograms. Panel **(a)**: Calibration curves for the nomograms at 1, 2, and 3 years. Panel **(b)**: DCA curves for the same time points. Panels **(c)** and **(d)**: Results of RT-qPCR, revealing that the expression levels of CD28 (*P* = 0.0140) and PF4 (*P* = 0.0021) were lower in the samples from patients with IPF

Analysis of the risk score across different clinical characteristics revealed that the risk score in the age subgroup of ≥ 75 years was higher than that in the < 75 years subgroup (Fig. 5a), and that older patients with IPF might be at higher risk of disease and poorer prognosis. Furthermore, univariate Cox regression analysis, the PH assumption test, and multivariate Cox regression analysis confirmed that the risk score serves as an independent prognostic factor (Fig. 5b). Based on the identified biomarkers, a nomogram was developed, indicating that higher total points corresponded to a lower survival rate for patients with IPF (Fig. 5c). The calibration curve’s slope for 1-, 2-, and 3-year predictions was close to 1, showing that the nomogram serves as a reliable predictor (Fig. 6a). Additionally, DCA indicated that the nomogram’s net benefit exceeds that of any single factor, highlighting its superior predictive performance (Fig. 6b). Moreover, reverse transcription quantitative polymerase chain reaction (RT-qPCR) results showed that both CD28 (*P* = 0.0140) and PF4 (*P* = 0.0021) were significantly downregulated in IPF samples (Figs. 6c-d). The downregulation of their expression may have been related to disease progression, inflammatory response modulation or fibrotic process, providing new clues for further investigation of the molecular mechanism of IPF.

### Biological pathways in high-risk and low-risk cohorts

**Fig. 7:**
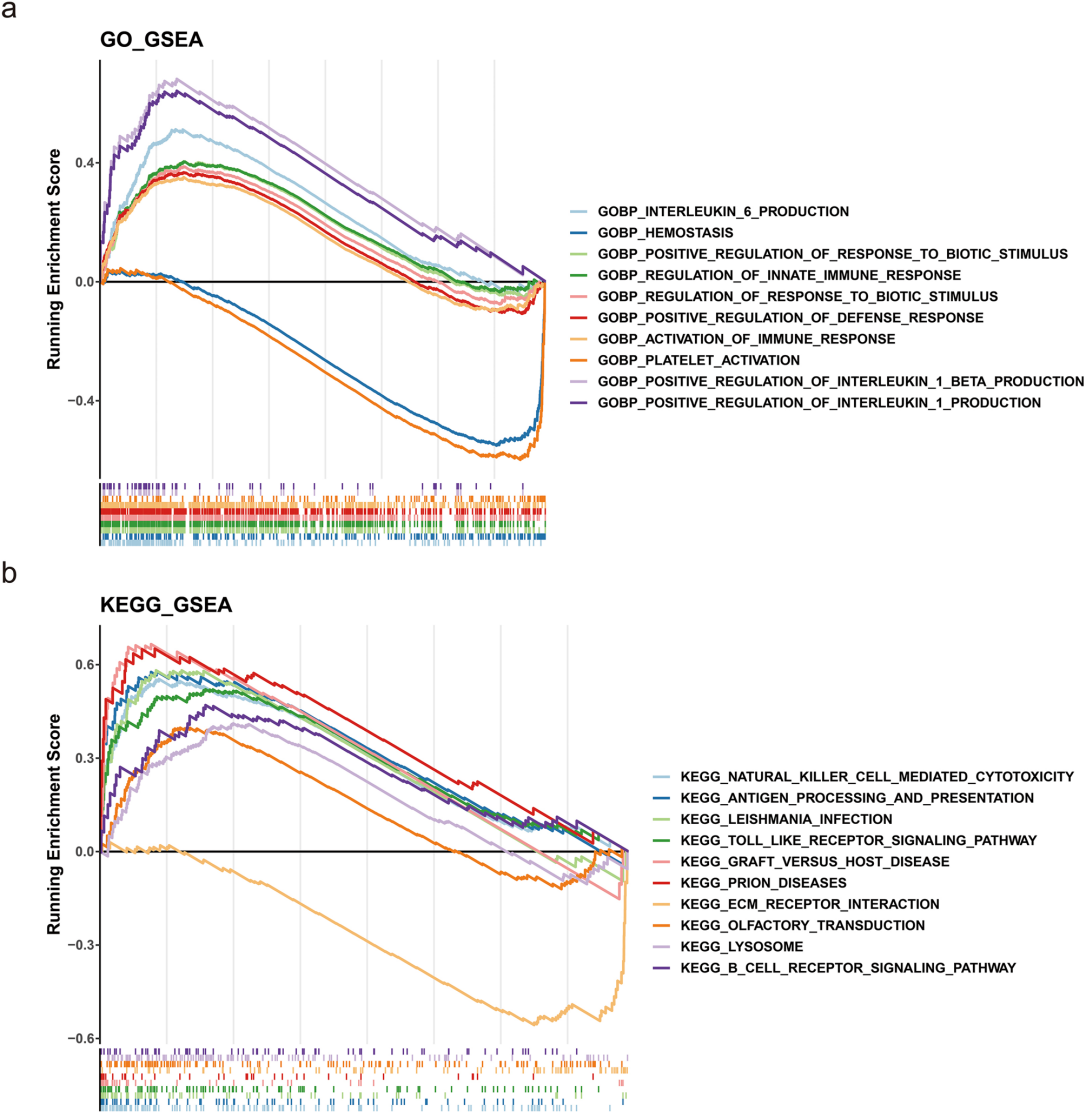
GSEA. Panel **(a)**: GO enrichment analysis highlighting differential biological pathways between high-risk and low-risk groups. Panel **(b)**: Differential KEGG enrichment analysis, comparing the pathways associated with the high-risk and low-risk groups

Gene Set Enrichment Analysis (GSEA) revealed differences in several biological pathways between the two risk cohorts. In terms of GO classifications, the high-risk cohort was primarily associated with positively regulated pathways, such as the positive regulation of interleukin-1β production and activation of the immune response. Conversely, pathways associated with negative regulation included hemostasis and platelet activation (Fig. 7a), suggesting that it may have been possible that wound healing was reduced in patients in the high-risk group for pulmonary fibrosis. The KEGG analysis revealed that positively regulated pathways encompassed prion diseases and graft-versus-host disease, while negatively regulated pathways involved extracellular matrix (ECM) receptor interactions (Fig. 7b). These findings suggested that biological processes in the high-risk cohort may have been more inclined to promote the activation of inflammatory responses and immune responses.

### Potential of immunotherapy in treating patients with IPF

**Fig. 8:**
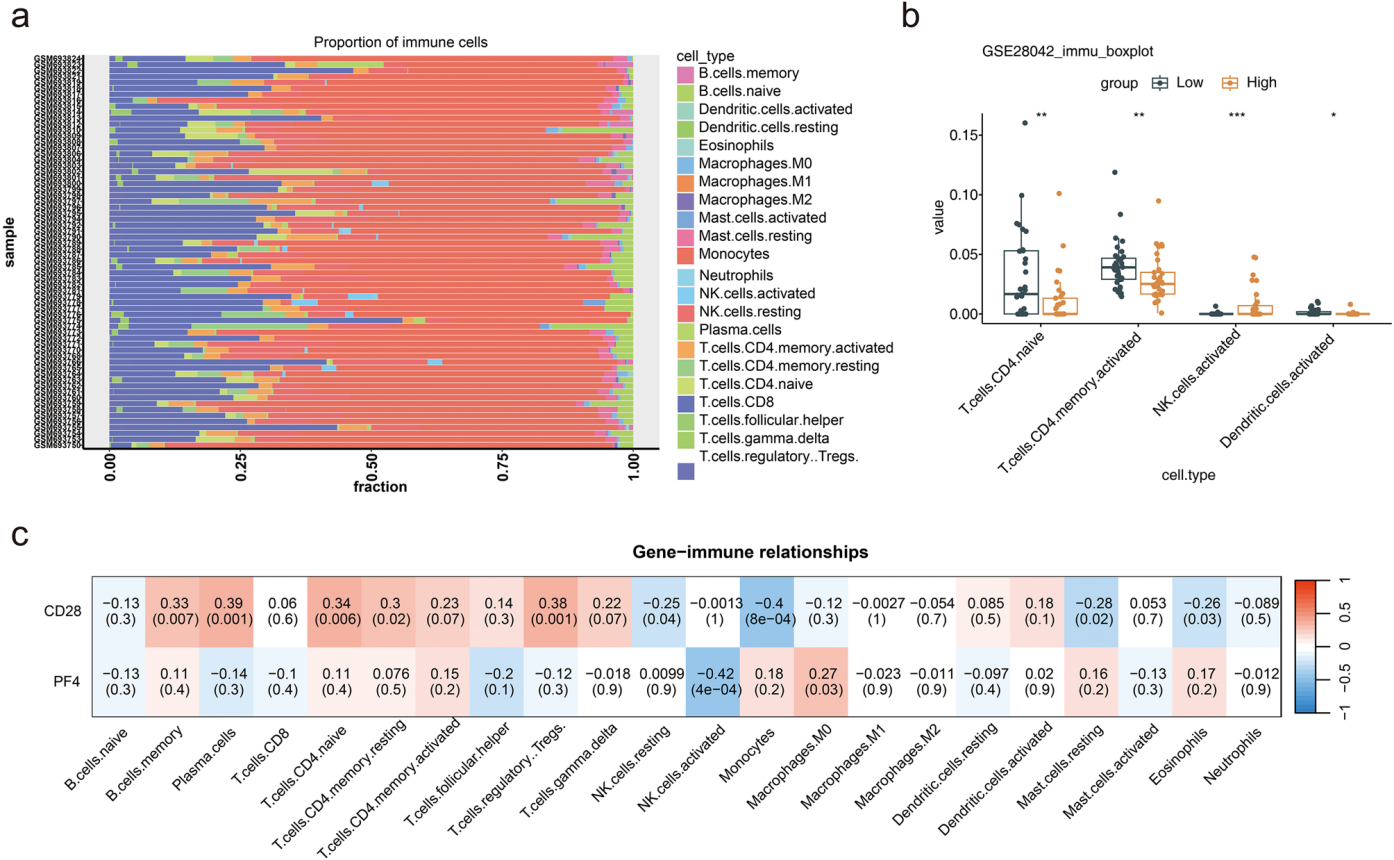
Immune correlation analysis concerning risk scores. Panel **(a)**: Proportion of 22 immune cell types infiltrating all IPF samples in the GSE28042 dataset. Panel **(b)**: Differential expression of immune cells between the high-risk and low-risk groups. Panel **(c)**: Correlation analysis between biomarkers and immune cell types

Immune infiltration analysis illustrated the proportions of 22 immune cell types present within the IPF samples (Fig. 8a). Four differential immune cell types were identified: naïve CD4 T cells, memory CD4 T cells, activated NK cells, and activated dendritic cells (Fig. 8b). Additionally, correlation analysis revealed that CD28 had a significant positive association with naïve CD4 T cells (correlation coefficient = 0.34, *P* < 0.01), while PF4 showed a negative association with activated NK cells (correlation coefficient = -0.42, *P* < 0.01) (Fig. 8c). In the immune microenvironment of IPF, the proportion and activity of specific immune cell types may have been regulated, thereby influencing disease progression, the degree of inflammatory response, and so forth.

### Regulatory network as a potential mechanism in IPF

**Fig. 9:**
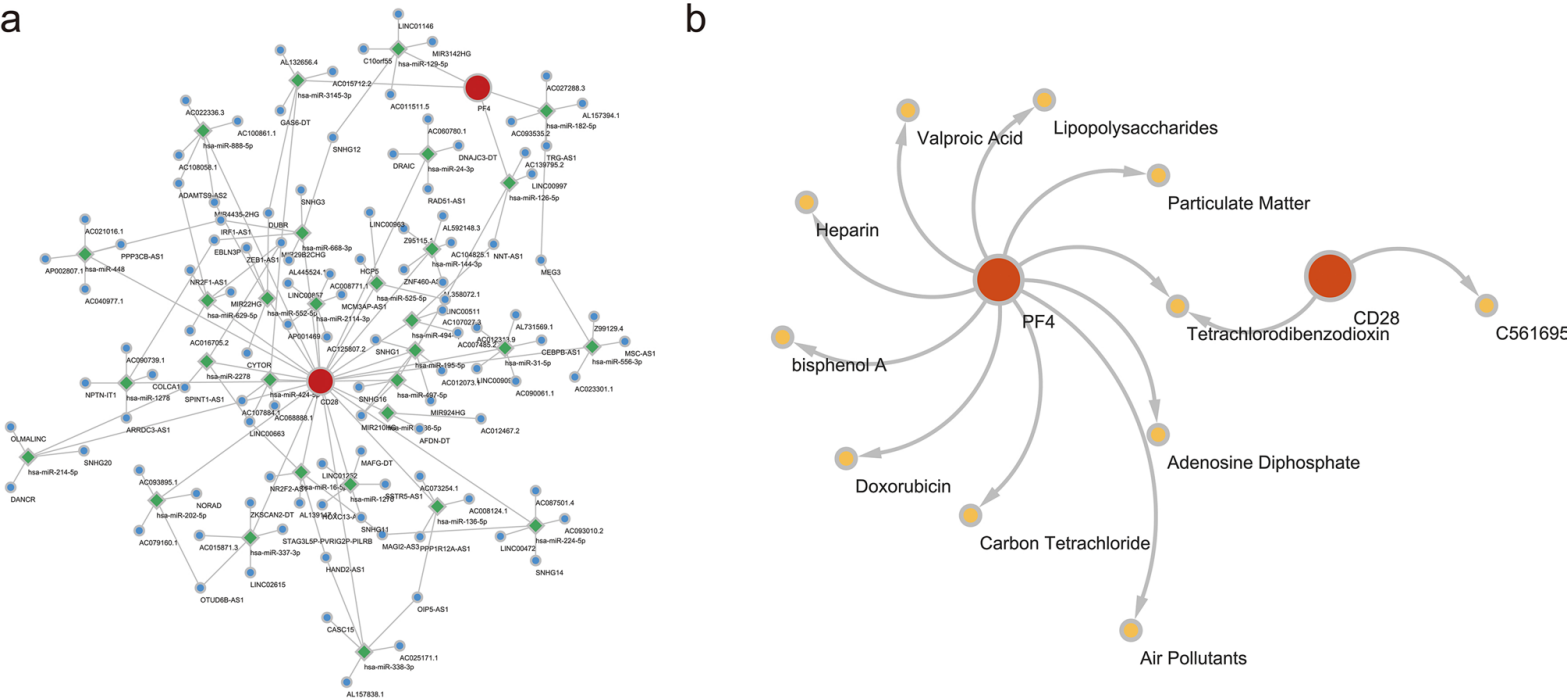
Competing endogenous RNA (ceRNA) network and drug prediction. Panel **(a)**: Illustration of the ceRNA network, where biomarkers are represented in red, miRNAs in green, and lncRNAs in blue. The lines connecting these components indicate the regulatory correlations among them. Panel **(b)**: Gene-drug relationship network, presenting biomarkers in red and their related drugs in yellow

By integrating miRNAs from the miRDB and TargetScan databases, a total of 26 miRNAs targeting CD28 and 4 miRNAs targeting PF4 were identified. Subsequently, 532 lncRNAs were predicted. Based on these findings, the lncRNA-miRNA-biomarker network was established, featuring interactions such as SNHG14-‘has-miR-224-5p’-CD28 and LINC01146-‘has-miR-129-5p’-PF4 (Fig. 9a). Additionally, a search of potential chemicals in the Comparative Toxicogenomics Database (CTD) yielded 11 chemicals, leading to the construction of a biomarker-chemical interaction network, including connections such as CD28-C561695 and PF4-tetrachlorodibenzodioxin (Fig. 9b).

## Discussion

IPF is characterized by its rapid progression and a complex pathogenesis. Increasing evidence suggests that PCD mechanisms, including cell proliferation, apoptosis, senescence, and autophagy, contribute significantly to the development of IPF. This study identified two biomarkers, CD28 and PF4, as predictors of prognosis in IPF through bioinformatics analysis and constructed a prognostic model. Although previous studies have found that significant downregulation of CD28 on circulating CD4 T cells is associated with poor prognosis in IPF patients, this study integrates multi-dimensional bioinformatics data, combining mitochondrial and programmed cell death-related genes, to construct a more comprehensive prognostic model. This not only provides new clues for the development of novel therapeutic strategies for the disease but also validates the reliability of previous research, further solidifying the status of CD28 as a prognostic biomarker for IPF.

Most T cells express cluster of differentiation 28 (CD28) on their surface, which is recognized as a distinctive marker for T cells and plays a crucial role in their activation pathways [36]. The involvement of immune responses in the pathogenesis of IPF remains a subject of debate. Notably, downregulation of CD28 expression has been reported in the peripheral blood of patients with IPF [37]. Recent studies indicate that a lower proportion of CD28 + T cells in the bloodstream correlates with poor prognosis and deterioration in lung function, as measured by Forced Vital Capacity (FVC) and Diffusion Lung Capacity for Carbon Monoxide (DLCO) [38]. Furthermore, diminished CD28 expression is associated with transplant-free survival and serves as a robust predictor of outcomes in this context [39]. Some investigations have proposed that targeting the CD28-mediated co-stimulatory pathway could represent a promising therapeutic strategy for preventing pulmonary fibrosis [40]. Consistent with these findings, our present study also identified decreased CD28 expression in IPF samples, suggesting that CD28 may serve as a potential therapeutic target that warrants further investigation.

Platelets are well known for their roles in coagulation and hemostasis. However, evidence supports their contribution to a diverse range of immunological processes [41]. Research has indicated that platelets may foster a hyperinflammatory environment by stimulating classical monocytes, which in turn produce various cytokines. These cytokines promote further platelet activation, a process linked to the exacerbation of lung damage and the progression of pulmonary fibrosis [42]. Human platelet factor IV (PF4), a heparin-binding protein released specifically by platelets, belongs to the chemokine family and exerts multiple effects, including hematopoiesis, angiogenesis inhibition, interference with platelet coagulation, promotion of the host inflammatory response, vascular suppression, and anti-tumor properties [43].

Recent studies have found that PF4 levels are elevated in fibrotic diseases such as systemic sclerosis, chronic liver fibrosis, cystic fibrosis, and myelofibrosis [44, 45]. However, there is a paucity of studies related to pulmonary fibrosis. Some studies have reported increased PF4 concentrations in bronchoalveolar lavage fluid (BALF) from individuals with scleroderma interstitial lung disease. Further research suggests that PF4 may play a critical role in radiation-induced pulmonary fibrosis (RIPF) and could be a potential target for treating RIPF [46]. Through bioinformatics analysis, our study shows for the first time that PF4 can serve as a prognostic marker, providing a new direction for the treatment of IPF.

Using GSEA, we found that pathways related to immunity are upregulated in the high-risk group, including those positively regulating the production of interleukin-1β (IL-1β), an effective pro-inflammatory mediator. Research by Marina R. Hadjicharalambous and colleagues has shown that a reduction in the inflammatory response in IPF fibroblasts is associated with weaker induction of inflammatory mediators by IL-1β, consistent with our findings regarding the upregulation of this pathway in the high-risk group [47]. Moreover, studies have indicated that persistent damage to alveolar epithelial cells can trigger chronic inflammation, characterized by infiltration of mononuclear macrophages and lymphocytes [48, 49]. Our results also indicate significant differences between the high-risk and low-risk groups in the activation of immune response pathways, with the high-risk group exhibiting notable upregulation.

ECM-receptor interactions involve the binding of extracellular matrix (ECM) molecules to receptors on the cell surface, facilitating signal transduction across the cell membrane. This process influences gene expression, phenotype, and behavior [50]. ECM-receptor interaction plays an important role in regulating processes such as cell migration, invasion, and epithelial-mesenchymal transition (EMT). Previous research has illustrated that ECM-receptor interactions are closely related to the regulation of IPF [51]. Our findings demonstrate that the ECM-receptor interaction pathway is upregulated in the high-risk group, further indicating its role in the regulation of IPF. Prior research suggests that Belumosudil (BLM) can be used to treat chronic Graft-Versus-Host Disease (cGVHD), IPF, liver damage, and other conditions. It can be inferred that cGVHD and IPF may share common therapeutic targets [52]. Our study also indicates notable differences between high-risk and low-risk groups in the Graft-Versus-Host Disease pathway. Additionally, our study is the first to identify differences in pathways related to hemostasis, platelet activation, and prion diseases between these risk groups.

Immune cells have been shown to play a key role in the onset and development of IPF [53]. In our study, we observed differences in immune cell profiles between the high- and low-risk groups, with a positive correlation between native CD4 T cells and CD28, and a significant negative relationship between PF4 and activated natural killer (NK) cells. The upregulation of programmed cell death-1 (PD-1) on CD4 T cells has been implicated in the production of inflammatory factors that contribute to lung fibrosis [54]. NK cells have the ability to spontaneously identify abnormal cells within the body and rapidly eliminate them through cytotoxic activity. They also produce various pro-inflammatory cytokines and chemokines, recruiting and activating other immune cells to initiate adaptive immune responses [55]. However,, it has been shown that NK cell activity and their overall content are diminished in patients with IPF [56]. The depletion of NK cell content has been identified as a prognostic indicator for progressive fibrotic interstitial lung disease [57]. Given these findings, targeting the genes CD28 and PF4 may provide a strategic approach to modulate immune cell expression in IPF, potentially delaying the onset and progression of the disease.

To identify potential therapeutic agents for IPF, we identified tetrachlorodibenzodioxin as a chemical that co-targets the two biomarkers, CD28 and PF4. Specifically, 2, 3, 7, 8-tetrachlorodibenzo-dioxin (TCDD) is an effective environmental pollutant and one of the major toxic components in cigarette smoke. It is involved in the regulation of xenobiotic metabolizing enzymes, particularly the cytochrome P450 1 family (CYP1), which is managed by aryl hydrocarbon receptors (AHR). The role of AHR, a unique cytochemical sensor, in tissue fibrosis has garnered attention in recent research. The migration and differentiation of lung fibroblasts are key events in pulmonary fibrosis. The AHR-ligand axis plays an important role in fibroblast migration and differentiation by enhancing the metabolism of arachidonic acid and inhibiting the increased expression of α-smooth muscle actin (α-SMA) induced by TCDD [58].

Currently, there is a lack of in-depth studies exploring the pathogenic correlation between TCDD exposure and IPF. In this study, we investigated the expression regulation and underlying mechanisms of MRGs and PCDRGs in patients with IPF. Two co-expressed genes identified within the database of human MRGs and PCDRGs were found to be involved in the response to TCDD. This suggests that TCDD may contribute to the pathogenesis of IPF by regulating genes associated with mitochondrial phagocytosis and programmed cell death. Given these findings, reducing TCDD exposure may be a key strategy for preventing the onset of IPF.

In this study, two biomarkers—CD28 and PF4—associated with IPF were selected through various analytical methods, including differential analysis, WGCNA, correlation analysis, univariate regression analysis, the PH hypothesis test, LASSO regression analysis, and multivariate regression analysis.

A risk model was constructed, categorizing patients into high- and low-risk groups based on the median value of risk scores. The evaluation and validation of the risk model demonstrated strong predictive performance. Additionally, the risk score was identified as an independent prognostic factor through univariate regression, the PH hypothesis test, and multivariate regression analysis, leading to the construction of a nomogram model. The nomogram model also exhibited robust predictive performance upon evaluation and validation.

Additionally, GSEA was conducted based on the stratification of high- and low-risk groups, revealing significant enrichment in pathways associated with immune response activation and hemostasis. Immune correlation analysis identified four immune cell types that differed between the high- and low-risk groups. Chemical predictions yielded a list of 11 chemical substances. These findings offer novel insights into the pathogenesis and prevention of IPF. Unlike previous prognostic models, we integrated multi-omics data, including genomics, proteomics, and metabolomics, to construct our prognostic model. This cross-omics data integration approach allows for a comprehensive understanding of the disease from multiple levels, providing richer and more accurate prognostic information compared to models that rely solely on a single omics dataset. Additionally, our prognostic model is closely linked to emerging targeted therapy decisions. In previous models, prognostic assessments rarely directly guided precise targeted therapy choices. Our model incorporates specific biomarkers to identify common targets, which have not been utilized in previous IPF treatments, representing an innovative attempt.

Disadvantages: This study has several limitations. Primarily, it relies on data obtained from public databases, which may present challenges related to varying collection standards and inconsistent data quality, potentially challenging the accuracy and consistency of the findings. Additionally, the limited sample size and insufficient diversity may impact the generalizability and representativeness of the results. To further elucidate the underlying mechanisms, experimental verification may be necessary, including biomarker validation and functional assessments.

## Conclusion

IPF is associated with a notably poor prognosis. This study identifies CD28 and PF4 as potentially critical factors in the pathogenesis of IPF, highlighting their influence on disease prognosis. These findings hold substantial potential for improving treatment strategies and prognostic assessments for patients with IPF in the future.

## Electronic supplementary material

Below is the link to the electronic supplementary material.

Supplementary Material 1

Supplementary Material 2

## Data Availability

All data generated or analysed during this study are included in this article. Further enquiries can be directed to the corresponding author.

## Abbreviations

PCD: Programmed cell death
IPF: Idiopathic pulmonary fibrosis
MRGs: Mitochondrial related genes
PCDRGs: PCD related genes
DEGs: The differentially expressed genes
WGCNA: Weighted Gene Co-expression Network Analysis
PH: Proportional hazards
RT-qPCR: Reverse transcription quantitative PCR
SASP: Senescence-associated secretory phenotype
GEO: The Gene Expression Omnibus
GO: Gene Ontology
KEGG: Kyoto Encyclopedia of Genes and Genome
PPI: Protein-protein interaction
LASSO: Least absolute shrinkage and selection operator
K-M: Kaplan-Meier
ROC: Receiver operating characteristic
AUC: Area Under Curve
DCA: Decision curve analysis
GSEA: Gene Set Enrichment Analysis
CTD: Comparative toxicogenomics database
RT-qPCR: The reverse transcription quantitative PCR
FVC: Forced Vital Capacity
DLCO: Diffusion Lung capacity for CO
PF4: Platelet factor IV
RIPF: Radiation-induced pulmonary fibrosis
IL-1β: Interleukin-1β
ECM: Extracellular matrix
EMT: Epithelial-mesenchymal transition
BLM: Belumosudil
cGVHD: Chronic Graft-Versus-Host Disease
NK: Natural killer
PD-1: The programmed cell death-1
TCDD: Tetrachlorodibenzo-dioxin
CYP1: The cytochrome P450 1
AHR: Aromatic hydrocarbon receptors
AhR: Aryl hydrocarbon receptors
α-SMA: α-smooth muscle actin
